# Assessment of Behavioral Approach and Behavioral Inhibition Systems in Mood Disorders

**DOI:** 10.32598/bcn.9.4.261

**Published:** 2018-07-01

**Authors:** Asghar Arfaie, Salman Safikhanlou, Abbas Bakhshipour Roodsari, Alireza Farnam, Ali Reza Shafiee-Kandjani

**Affiliations:** 1. Research Center of Psychiatry and Behavioral Sciences, Tabriz University of Medical Sciences, Tabriz, Iran.; 2. Department of Psychiatry, Faculty of Medicine, Tabriz University of Medical Sciences, Tabriz, Iran.; 3. Department of Psychology, Faculty of Education & Psychology, Tabriz University, Tabriz, Iran.; 4. Road Traffic Injury Research Center, Tabriz University of Medical Sciences, Tabriz, Iran.

**Keywords:** Bipolar mood disorder, Behavioral brain system, Major depression, Behavioral Approach System (BAS), Behavioral Inhibition System (BIS)

## Abstract

**Introduction:**

Psychiatric disorders could be evaluated in terms of behavioral activation and inhibition systems. Dysregulation of these systems may lead to development of manic or depressive episodes in patients with mood disorders. This study aimed to identify Behavioral Approach System (BAS) and Behavioral Inhibition System (BIS) hypersensitivity as the functional brain system behaviors in patients with major depressive disorder and bipolar mood disorder I, compared to healthy individuals.

**Methods:**

This case-control study was conducted in Razi Psychiatric Hospital, a mental health referral center in Northwest of Iran. The study consisted of two groups of patients, one with major depressive and the other with bipolar mood disorders and one healthy group. Each group had 40 patients (20 men and 20 women). The study data were collected through BIS and BAS questionnaire, Beck Depression Inventory (BDI-II), Young Mania Rating Scale (YMRS). The obtained data were analyzed by SPSS version 18.

**Results:**

The findings showed a significant negative correlation between BIS, BAS and BAS subscales with the severity of depression and positive correlation with mania symptoms (P<0.05).

**Conclusion:**

BAS and BIS dysregulations may predispose people to mood disorder symptoms. BAS is hyperactive during manic phase and may predict the symptom severity of bipolar mood disorder.

## Highlights

- Patients with bipolar disorder had higher BAS, while those with MDD scored lower in BAS scales compared to healthy people.
- BAS-drive had the highest score in bipolar patients and the lowest score in MDD patients, but BAS-reward responsiveness was the highest in patients with bipolar disorder and lowest in those with depression.
- Bipolar patients scored higher in BAS-fun-seeking, BAS-drive and BAS-reward responsiveness, while patients with major depression had lower scores on BAS-FS, BAS-D, and BAS-RR subscales compared to the healthy individuals.

## Plain Language Summary

Dysregulation of behavioral activation and inhibition systems may develop into manic or depressive episodes in patients with mood disorders. In this study, we tried to identify Behavioral Approach System (BAS) and Behavioral Inhibition System (BIS) hypersensitivity as the functional brain system behaviors in patients with major depressive disorder and bipolar mood disorder I compared to healthy individuals. The study consisted of two groups of patients; one with major depressive and the other with bipolar mood disorders as well as one healthy group. Each group had 20 men and 20 women. The study data were collected through BIS and BAS questionnaire, analyzed with Beck depression inventory, Young mania rating scale in SPSS.

The findings showed a significant negative correlation between BIS, BAS and BAS subscales with the severity of depression and positive correlation with mania symptoms. BAS and BIS. Dysregulations may predispose people to mood disorder symptoms. BAS is hyperactive during manic phase and may predict the symptom severity of bipolar mood disorder.

## 1. Introduction

Currently, millions of people are affected by mental disorders worldwide. This problem results in huge economic and social costs due to the burden of disease itself and damage to the quality of life. Major Depressive Disorder (MDD) and Bipolar Mood Disorder (BMD) are serious mental illnesses which negatively impacts a variety of important life aspects. In this regard, the lifetime prevalence of depression and BMD are 17% and 3% in the general population that put them in the fourth and sixth highest ranks in the global disease list, respectively. In addition, after ischemic heart disease, depression is the second most prevalent mental condition. It is notable that based on the report of the World Health Organization, bipolar disorder has remained among the top 10 causes of years lived with disability worldwide (Murray & Lopez, 1996).

Biopsychological theory of personality was introduced by Gray (1977; 1981). Based on this theory, the Behavioral Approach System (BAS) is associated with reward sensitivity while Behavioral Inhibition System (BIS) is related to avoidance or sensitivity to punishment (Gray, 1977; Watson, Wiese, Vaidya, & Tellegen, 1999).

The systems are suggested to integrate biological and environmental factors with special focus on personality (Gray, 1982; McNaughton & Corr, 2004). They reflect the different types of reinforcements of two biological broadband (activation/inhibition) motivational systems. These systems are hypothesized to regulate approach and withdrawal behavior in response to environmental cues (Gray, 1982).

Whereas activation of BIS is supposedly decrease the individual’s behavior toward stimuli such as threat, punishment, novelty, and frustrate non-reward cues, BAS increases one’s approach toward stimuli sensitive to reward and non-punishment. Gray and other researchers (Carver & White, 1994; Tomarken & Keener, 1998; Watson, Wiese, Vaidya, & Tellegen, 1999) proposed that BAS is associated with negative mood states, and controls positive affectivity that refers to goal-seeking behavior in response to reward cues.

BIS governs anxiety and may control negative affectivity in response to cues of threat. Originally, Gray’s model was applied to anxiety, but Depue and Iacono (1989) and others (Tremblay, Naranjo, Cardenas, Herrmann, & Busto, 2002; Pinto-Meza, et al., 2006) focused on a broad range of psychological distress and mental problems with the constellations of BIS and BAS scales and subscales. Different personalities may show various degrees of responsiveness. Evidence from increasing goal-seeking behavior and BAS activation or fearlessness and BIS inactivity during the manic phase of BMD appear to link the BIS/BAS scales with MDD and BMD (Carver & White, 1994; Meyer, Johnson, & Winters, 2001).

Several studies have shown an association between bipolar disorder and depression as a spectrum (Meyer, Johnson, Carver, 1999; Johnson, Turner, & Iwata, 2003). Other studies have mentioned that people’s stable personality traits are congruent with their emotional tone (Rusting, 1998).

According to this theory, symptoms of manic and depressive patients relate to neurotransmitters that underlie BIS and BAS, where mania results from an excessive dopamine-mediated responsiveness to rewards correlated with high BAS, while depression results from defective serotonin- and norepinephrine-mediated response to threats with low BIS. Also, most studies in cognitive neuroscience suggest that BIS scales, when measured with questionnaires, positively correlate with negative affects, while BAS scales positively correlate with positive affects (Jones, Mansell, & Waller, 2006). Additionally, it has been shown that self-reported BAS sensitivity can predict the occurrence and the severity of MDD in an 8-month follow-up (Kash, Rottenberg, Arnow, & Gotlib, 2002).

Understanding the role of personality underpinnings and behavioral brain system in mood disorders may help clinicians to predict the potential symptom development and establish preventive or treatment interventions. The major objective of the current study was to explore BAS and BIS implications in mood disorders. Moreover, we aimed to identify the potential biomarkers and experimental paradigms in the early intervention based on self-reported BIS and BAS functioning in individuals with mood disorders.

## 2. Methods

### 2.1. Setting and participants

In this case-control study, the subjects were recruited from Razi Psychiatric Hospital in Tabriz, Iran, from June 2013 to July 2014. We employed a simple random sampling method to recruit participants in two groups of patients, one with MDD (without psychotic features) and one with BMD I (with acute psychotic symptoms) based on DSM-IV-TR criteria. The third group consisted of healthy individuals without a history of psychiatric disorders. Twenty male and 20 female participants aged 18–65 years were recruited for each group. Those who did not fully complete the self-reported questionnaires were excluded from the study.

The study was explained to the participants and a written informed consent was obtained from them. All people were recruited based on an individual code given by a psychiatrist. Both participants and the examiners were blind to the experimental design.

### 2.2. Research instruments

#### 2.2.1. Behavioral approach system/Behavioral Inhibition system scales

The scales were of 4-point Likert-type questionnaires scored from 1 to 4 (1=Strongly disagree to 4=Strongly agree) consisting of 24 items, where items from the original BIS/BAS scales were modified based on Azari Language. To measure BIS, we included 7 items related to anticipation of punishment, while for measuring the BAS (which has three subscales of its own) we included five items about anticipation or occurrence of the reward, Reward Responsiveness (RR); four items about pursuit of desired goals for Drive (D); four items about desires, Fun-Seeking (FS) for new rewards and impulsive approach to potential.

The internal consistency of the BIS subscales was determined based on Moazzen, Azad Fallah, and Safi’s (2009) study that was 0.74. And for the three subscales of BAS, they were 0.73, 0.76 and 0.66, respectively. In our study (N=120), the Cronbach α value was 0.66 for the state BIS, and 0.89 for the state BAS. It was 0.70, 0.64 and 0.61 for RR, D, and FS or response to the BAS subscales, respectively.

#### 2.2.2. Revised Beck Depression Inventory (BDI-II)

Revised Beck Depression Inventory is a screening self-reported instrument containing 21 items, which provides a highly reliable assessment regardless of the study population (Beck, Steer, & Garbin, 1988). Farsi version of BDI-II was released with high validity and reliability (Ghassemzadeh, Mojtabai, Karamghadiri, & Ebrahimkhani, 2005).

#### 2.2.3. Young Mania Rating Scale (YMRS)

Young Mania Rating Scale is an 18-item questionnaire to measure the severity of manic symptoms such as irritability, disruptive and aggressive behavior, elevated mood, hyperactivity, impaired language and or thinking and other signs based on interpretation of scores from this scale. The scores were classified as the following: 0–9, without mania; 10–17, hypomania; 18–21, manic border; 22–35, mild to moderate mania; 36–53, moderate to severe mania; and finally, 54 or higher, severe mania. It has been standardized in Persian language (Barekatain, Tavakoli, Molavi, Maroufi, & Salehi, 2007).

#### 2.2.4. Structured Clinical Interview for DSM-IV Axis I Disorders (SCID-I)

We used Structured Clinical Interview (SCID-I), as a tool for the diagnosis of disorders based on the Diagnostic and Statistical Manual of Mental Disorders (DSMIV), fourth edition. Reliability for the SCID has been shown to be strong. Also, the Farsi version of the tests achieved a high rate of agreement (kappa) for SCID used for the diagnostic decision and research approach.

#### 2.2.5. Demographic characteristics

In this study, the patients’ age, gender, marital status, education, occupation, patient records, treatments and hospitalizations were collected and the relationship between BAS/BIS and MDD and BMD was explored.

### 2.3. Data analysis

The sample size was estimated as 40 for each group (In total 120 participants) based on Alloy and colleagues (Alloy et al., 2008) study data with a significance level of α=0.05 and power of 80% and mean of 17.03±1.97 for BAS-RR and accepting difference as 1.5 unit. We assessed each group by means of a set of BIS and BAS, BDI-II, YMRS scales as well as scales related to the BAS-RR, and analyzed the data in SPSS version 18, by performing descriptive statistics, MANOVA, Pearson correlation coefficient significance test and multiple regressions. All tests were 2-tailed at significance level of 0.05.

## 3. Results

Initial analyses were conducted to calculate descriptive statistics. Table 1 presents basic sociodemographic characteristics. Then, we compared BIS/BAS levels in mood disorder groups with a normative sample. Basic correlations between the BIS/BAS levels were evaluated. The mean and standard deviations are presented sex- and group-specific (Table 2).

**Table 1.** Sex -and group-specific presentation of demographic and clinical characteristics

**Cases (N)**		**Healthy**	**BMD**	**Depression**	**Total**
Sex	Male	20	20	20	60
	Female	20	20	20	60
Education	Primary	2	4	4	10
	Secondary	8	10	21	39
	High school	18	19	17	54
	University	6	5	6	17
Marital status	Single	11	9	9	29
	Married	34	15	3	85
	Divorced	0	5	1	6
Job	Self-employed	0	5	2	7
	Employed	12	3	4	19
	Unemployed	15	14	19	48
	Retired	0	2	0	2
	Student	0	1	0	1
	Homemaker	13	15	15	43

**Table 2.** Group-specific means and standard deviations for scales and sub-scales

**Groups**	**Total**		**MDD**		**BMD**		**Healthy**	
	
**Scale and Sub-Scales**	**Mean**	**SD**	**Mean**	**SD**	**Mean**	**SD**	**Mean**	**SD**
BIS	20.57	5.07	26.35	1.66	15.68	2.24	19.68	3.32
BAS	33.76	11.83	20.35	4.73	47	3.06	33.93	5.60
BAS-FS	10.03	3.47	6.18	1.38	13.55	1.65	10.38	2.01
BAS-D	10.90	3.97	6.43	1.60	14.95	1.32	11.33	2.49
BAS-RR	12.83	5.29	7.75	2.72	18.50	1.57	12.23	3.96
BDI-II	19.64	23.51	52.28	4.50	0.50	0.82	6.15	3.34
YMRS	25.29	28.99	4.63	2.58	65.48	7.92	5.78	3.23

The results revealed a significant difference between the three groups in terms of BAS/BIS activity levels. Based on the regression model, BIS and BAS could predict depression with its related positive and negative intense impacts. They also could predict depressive episodes in three groups of the participants. BAS-RR subscale is a negative predictor for bipolar disorder and major depression, while in healthy participants, we did not find any significant relationship.

BAS seems to be a significant predictor of mania symptoms’ intensity. The results obtained from the FS subscale in bipolar patients showed a significant predicting value but among individuals with major depression, we could not find any significant relationship. As shown in Table 3, there are significant correlations between BIS and severity of manic symptoms, also between BAS and BAS subscales (BAS-RR, BAS-D, BAS-Fun) with the severity of depression. In addition, significant relationships between BIS and severity of depression and BAS with severity of mania symptoms were detected (P<0.005).

**Table 3.** Correlations between variables

**Variables**		**BIS**	**BAS**	**BAS-FS**	**BAS-D**	**BAS-RR**	**BDI-II**	**YMRS**
BIS	Correlation	1	−0.76	−0.77	−0.72	−0.65	0.84	−0.68
	P		0.000	0.000	0.000	0.000	0.000	0.000
BAS	Correlation	−0.76	1	0.90	0.94	0.94	−0.85	−0.80
	P	0.000		0.000	0.000	0.000	0.000	0.000
BAS-FS	Correlation	−0.77	0.90	1	0.82	0.75	−0.82	0.74
	P	0.000	0.000		0.000	0.000	0.000	0.000
BAS-D	Correlation	−0.72	0.94	0.82	1	0.81	−0.83	0.73
	P	0.000	0.000	0.000		0.000	0.000	0.000
BAS-RR	Correlation	−0.65	0.94	0.75	0.81	1	−0.74	0.78
	P	0.000	0.000	0.000	0.000		0.000	0.000
BDI-II	Correlation	−0.84	−0.85	−0.82	−0.83	−0.74	1	−0.58
	P	0.000	0.000	0.000	0.000	0.000		0.000
YMRS	Correlation	−0.68	0.80	0.74	0.73	0.78	−0.58	1
	P	0.000	0.000	0.000	0.000	0.000	0.000	

## 4. Discussion

Patients with bipolar disorder had higher BAS, while those with MDD scored lower in BAS scales compared to healthy people. Meanwhile, we noticed that the highest and lowest activity levels in the BAS-FS subscale were shown in BMD and MDD, respectively. These results are in line with the studies conducted by Pinto-Meza et al. (2006) and Alloy et al. (2008). BAS-D had the highest score in bipolar patients and the lowest score in MDD, but BAS-RR was the highest in patients with bipolar disorder and lowest in those with depression which was also previously revealed (Henriques & Davidson, 1990). Bipolar patients scored higher in BASFS, BAS-D and BAS-RR, while patients with major depression had lower scores on BAS-FS, BAS-D and BAS-RR subscales compared to the healthy individuals. The results are in agreement with the findings of most recent studies (Quilty, Mackew & Bagby, 2014; Fletcher, Parker, & Manicavasagar, 2013).

The negative correlation between BAS and MDD suggests that these individuals have deficits in controlling desires, goal-seeking behavior, and positive emotions. Therefore, anhedonia in depressed individuals is justified by a low activity level of BAS. We also found a positive correlation between BIS and depression which suggests that the patients with depression had sensitivity and hyperactivity in BIS leading to negative emotions. The results of some published works are in agreement with the current study (Kash et al., 2002), meanwhile a few studies disagree with our results (Meyer, Johnson, & Carver, 1999; Jones, Mansell, & Waller, 2006).

Our findings suggest that BAS is hyperactive in bipolar disorder which supports the dysregulation in these patients. An inverse relationship between BAS, RR and depression suggests a BAS dysregulation in depressive patients. Depressed patients had lower responsiveness to reward and receive little reward as a motivation. While all patients scored higher on BIS in comparison with normal individuals, patients with BMD scored higher on BAS scales compared to patients suffering from depression. Our study had some limitations too. Small sample size, inability to discontinue medications, and self-reporting nature of BAS/BIS scales all constitute major limitations of this study. Dysregulations of BAS and BIS may predispose affected individuals to mood disorder symptoms. Our study findings emphasize the role of the behavioral brain systems as integrated models of biopsychological aspects in the occurrence and continuation of mood disorders. Any problems in these systems or related domains may predict the development of mood disorders.

## Ethical Considerations

### Compliance with ethical guidelines

The study was conducted based on regional ethical guidelines. Informed consents were taken and all participants were free to leave the study whenever they would like to do.
